# Securing federated learning with blockchain: a systematic literature review

**DOI:** 10.1007/s10462-022-10271-9

**Published:** 2022-09-16

**Authors:** Attia Qammar, Ahmad Karim, Huansheng Ning, Jianguo Ding

**Affiliations:** 1 School of Computer and Communication Engineering, University of Science and Technology Beijing, Beijing, China; 2 Department of Information Technology, Bahauddin Zakariya University, Multan, Pakistan; 3 Department of Computer Science, Blekinge Institute of Technology, Karlskrona, Sweden

**Keywords:** Federated learning, Blockchain, Security, Privacy, Blockchain-based FL, Systematic literature review

## Abstract

Federated learning (FL) is a promising framework for distributed machine learning that trains models without sharing local data while protecting privacy. FL exploits the concept of collaborative learning and builds privacy-preserving models. Nevertheless, the integral features of FL are fraught with problems, such as the disclosure of private information, the unreliability of uploading model parameters to the server, the communication cost, etc. Blockchain, as a decentralized technology, is able to improve the performance of FL without requiring a centralized server and also solves the above problems. In this paper, a systematic literature review on the integration of Blockchain in federated learning was considered with the analysis of the existing FL problems that can be compensated. Through carefully screening, most relevant studies are included and research questions cover the potential security and privacy attacks in traditional federated learning that can be solved by blockchain as well as the characteristics of Blockchain-based FL. In addition, the latest Blockchain-based approaches to federated learning have been studied in-depth in terms of security and privacy, records and rewards, and verification and accountability. Furthermore, open issues related to the combination of Blockchain and FL are discussed. Finally, future research directions for the robust development of Blockchain-based FL systems are proposed.

## Introduction

Federated Learning (FL) was first introduced by Google as a distributed machine learning paradigm to train the model with local data from devices while ensuring privacy (McMahan et al. 2017). A couple of devices are participated to build the FL model locally. The trained local model updates are sent to the central FL server and aggregated to optimize the global FL model. Compared to conventional machine learning, FL protects the data of clients and prevents the disclosure of local data privacy. The data used for model training are broadcasted from various participating companies and users to converge the FL model. They have the right to improve the quality of model updates and can reduce the model performance. Furthermore, FL is remarkably used in real-world applications in particular healthcare, finance, transportation, and smart cities, to mention a few. (Xu et al. 2020; Chen et al. 2020; Long et al. 2020; Tan et al. 2020; Zheng et al. 2021). Although FL outperforms and shows its effectiveness as preserving privacy by design, optimized bandwidth, and low latency. However, FL endures various limitations in terms of security and privacy. The model parameters aggregation scheme implemented in FL, makes the entire model reliant on the central FL server. The failure of a central server leads to Single Point of Failure (SPoF) and Distributed Denial of Service (DDoS) attack. Furthermore, in the current FL system, there is no transparent mechanism to record the local model updates. Hence, an effective decentralized system is required to detect and prevent malicious updates. The aforementioned attacks can be solved through the integration of blockchain technology into federated learning systems. Blockchain has an ability to cope with these challenges, ensure decentralized storage of model updates and traceability of the model. Furthermore, blockchain follows the combination of chain, tree, and graph structure to make it temper-proof and record history. Similarly, participated clients are verified and send the model updates, maintaining the order of blocks consistent and immutable. With the addition of a digital currency, blockchain has great potential to attract participants of model training (Toyoda and Zhang 2019). At the same time, blockchain has introduced immutability of records through consensus algorithms such as Proof-of-Work (PoW). Therefore, consensus and incentive schemes are wisely implemented, which successfully motivate the communication of data in FL. The incentives or rewards are provided equally to the size of contribution in the FL model training process. In literature, several studies are available related to federated learning (Cheng et al. 2020; Li et al. 2020b; Abdulrahman et al. 2021), blockchain technology (Andoni et al. 2019; Agbo et al. 2019; Ali et al. 2020; Wang et al. 2019b), and blockchain-based federated learning approaches (Drungilas et al. 2021; Shayan et al. 2021; Cui et al. 2021; Qu et al. 2021; Chai et al. 2021; Hua et al. 2020). Li et al. (2021a) discussed the blockchain-based federated learning (BCFL) architecture with respect to types, design, model improvement, and incentive mechanism. However, there is a lack of systematic literature review (SLR) on the combination of Blockchain and FL approaches considering the factors of security, incentive mechanism, attacks detection, attack defense, etc. In this systematic literature review paper, we explore the blockchain-based FL techniques from the year 2016–2022, discussing the existing federated learning issues, blockchain-based federated learning architecture, contemporary approaches, potential challenges integrating the Blockchain with FL, and the future directions. Comprehensively, the main contributions are highlighted below:

1. A thorough literature review to identify security vulnerabilities in FL is conducted and which approaches are most suitable, and concluded that introducing the blockchain technology into FL provides a much more secure solution.
2. An overview of federated learning and blockchain technology with its working mechanism is provided. In addition, the existing issues in FL that can be solved by integrating blockchain with FL are elaborated in detail.
3. The blockchain-based federated learning architecture, its entire workflow, and blockchain deployment frameworks implemented in federated learning are investigated.
4. The state-of-the-art blockchain-based federated learning approaches are presented, in the context of security and privacy, record and reward, and verification and accountability.
5. Based on a deep analysis, the outstanding challenges of integrating Blockchain into federated learning are discussed along with their downsides.
6. Finally, to improve the practicality of the blockchain-based federated learning systems, future directions are suggested.

The remainder of this systematic literature review paper is organized as follows. In Sect. 2 discusses the research method of a systematic review with research findings and questions. Section 3 provides an overview of federated learning and blockchain technology. Section 4 presents the integration of blockchain into federated learning with its architecture and workflow. Section 5 discusses the state-of-the-art: securing federated learning with blockchain approaches. Section 6 provides the discussion on SLR results. Section 7 introduces the open issues and future directions. Finally, Sect. 8 concludes the paper.

## Research method of the systematic review

A systematic literature review (SLR) has an objective to identify, assess and analyze all available research studies in a certain area of interest. An SLR must be completed using a thorough search strategy that is impartial and fair. The search strategy must guarantee a comprehensive search for evaluations. At the time of this paper, no SLR provided a meticulous review of blockchain-based federated learning. This paper aims to fill this gap by conducting an SLR following Kitchenham’s methodology (Kitchenham 2004).

### Search process

**Fig. 1:**
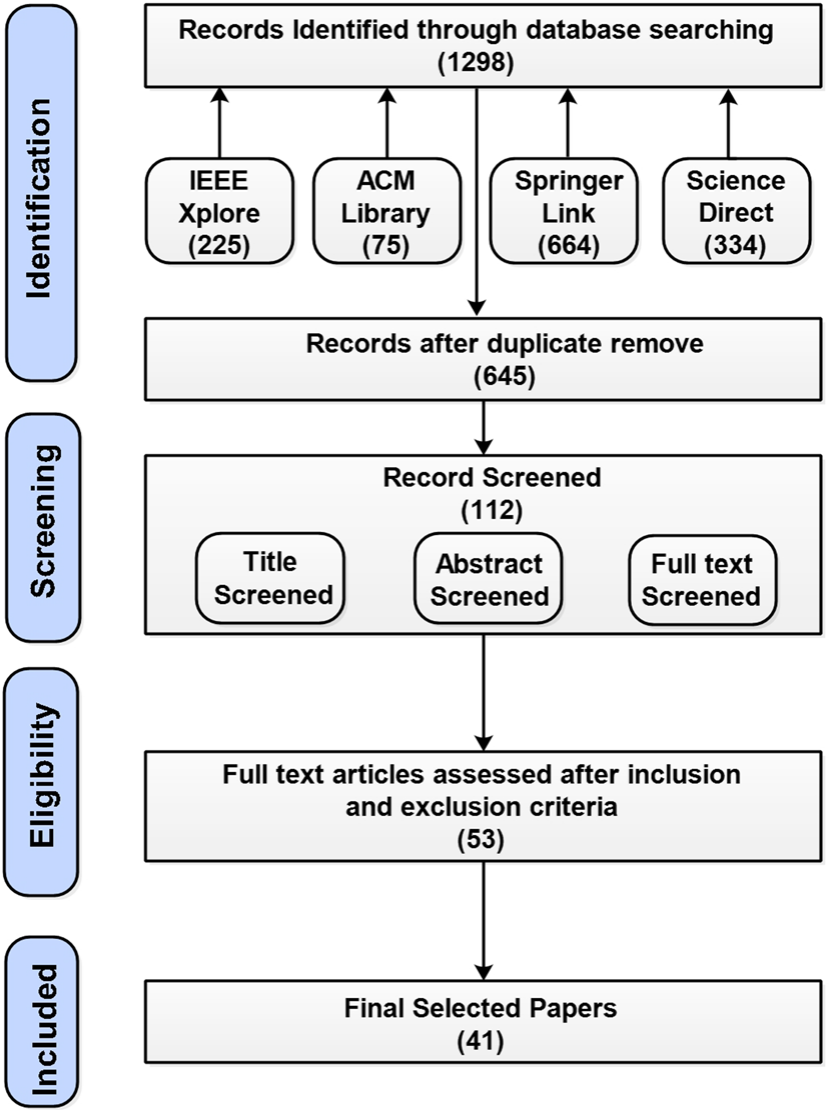
PRISMA flow diagram of the systematic review phases. Adapted from (Moher et al. 2009)

In this SLR, the studies were explored from published as well as archive repositories to highlight the trend of blockchain-based federated learning in academia. In Fig. 1, PRISMA (Preferred Reporting Items for Systematic Reviews and Meta-Analyses) flow diagram is presented (Moher et al. 2009).

**Fig. 2:**
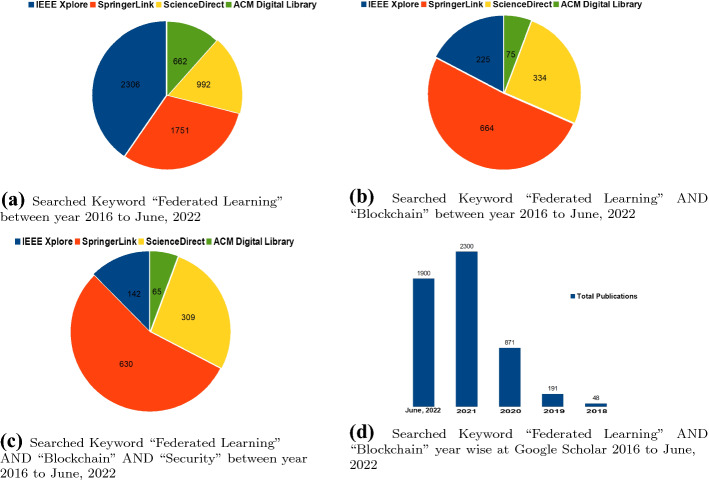
Publications in heterogeneous databases

The number of publications is filtered out at each stage and the terms “security”, “privacy”, “rewards”, “record”, “accountability”, and “auditing” were considered crucially in each study. The stages of the PRISMA flow diagram were divided into four parts: (1) identification, where 1298 records were provided from heterogeneous databases such IEEE Xplore, ACM Digital Library, SpringerLink, and ScienceDirect between the years 2016 to June 2022; (2) in the screening stage, the filter was applied based on the title, abstract, and full text; (3) for eligibility 53 papers were considered based on the inclusion and exclusion criteria and (4) finally, the included studies were presented. After several checks and screenings, 41 research papers were selected for this SLR.

The keyword “Federated Learning” in the aforementioned databases was searched as depicted in Fig. 2a. The result shows that most of the studies were published in IEEE Xplore and a few in ACM Digital Library. The search terms were combined into a search string using the conjunction (AND) operator to retrieve the exact studies. The other search keywords were used in the databases like “Federated Learning” AND “Blockchain” as presented in Fig. 2b, where most of the studies were published in SpringerLink. Similarly, the search string such as “Federated Learning” AND “Blockchain” AND “Security” was searched and the result shows the highest publication ratio in SpringerLink as depicted in Fig. 2c. Moreover, a year-wise trend of the keywords “Federated Learning” AND “Blockchain” at Google Scholar is presented in Fig. 2d. Consequently, in the year 2021 total number of publications was highest as compared to previous years.

### Inclusion and exclusion criteria

This SLR provides the readers with a clear understanding of blockchain-based federated learning approaches and an in-depth description of terms related to security, privacy, records, rewards, accountability, and characteristics of blockchain over FL. Hence, for this purpose, the inclusion and exclusion criteria were adopted as presented in Table 1. The research studies between the years 2016 to June 2022 were included in this SLR because contemporary information is available during this period. Furthermore, duplicates and papers in other languages were excluded with justification.

**Table 1:** Inclusion and exclusion criteria with justification

Criteria		Justification
Inclusion	Studies published online in years 2016 to June 2022.	The fundamental research on this topic has been revealed in the papers published in recent year
	Studies based on the integration of blockchain and federated learning	Have promising research status in academia and industry
	Papers that address the mechanism of blockchain-based federated learning in context of (1) security and privacy, (2) record and reward and, (3) verification and accountability approaches as it leads to a secure FL system	Have an auspicious research status in academia and industry
Exclusion	Papers that were not written in English	No ability to examine non-English language papers
	Duplicate material from a similar study	Novel research papers were considered and repetitive information was removed
	Short research papers of less than 4 pages	These studies did not provide much knowledge, therefore excluded from our research

### Research questions

In this SLR, a structured and comprehensive overview of all related studies in the context of blockchain-based federated learning is presented. The five Research Questions (RQs) are addressed as below:

#### RQ1

What are the potential security and privacy attacks in traditional federated learning which can be solved by blockchain technology?

#### RQ2

What are the promising characteristics of blockchain for federated learning to provide a secure environment?

#### RQ3

What are the state-of-the-art blockchain-based federated learning approaches in security and privacy, records and rewards as well verification and accountability to secure the traditional FL system?

#### RQ4

What are the research challenges in the implementation of blockchain-based federated learning and how can it bring new issues?

#### RQ5

What are promising future research directions for effectively implementing the blockchain technology in federated learning?

## Federated learning and blockchain

### Overview of federated learning

**Fig. 3:**
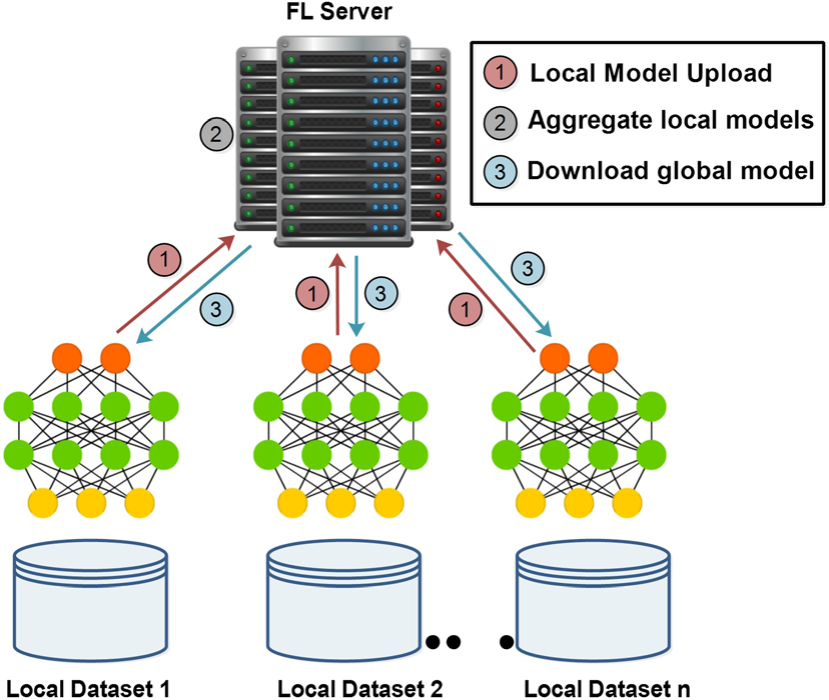
Federated learning architecture

Federated learning (McMahan et al. 2017) is defined as a centralized training mechanism that ensures user privacy by sharing unique data distribution properties. The clients (FL participants) upload the training data as model updates to the FL server, based on their private local datasets. Afterward, the FL server aggregates the local model updates and builds the global model for users to download (see Fig. 3).

In Eq. (1), in each training round *t*, the FL server sends the initial model updates to the selected FL participants $m_t$. Then the selected FL clients update their local model at their end and apply an initial model to train the local data. Each FL participant uploads the local model updates to the central FL sever, which then accumulates and converts them into a global model.

1 $$\begin{aligned} G_{t+1} = G_t + \frac{1}{m_t} \sum _{k=1} ^ {m_t} l_t^k, \end{aligned}$$

Here, $G_t$ represents the current global model in the $t$th iteration, whereas $G_{t+1}$ denotes the fully converged global model. The $l_t^k$ denotes local model uploads by the $k$th FL participant. The FL revolves around the aggregation algorithm which is called vanilla Federated Average (FedAvg) to enable the accumulation of the local model updates. The generalization and re-parametrization of the FedAvg algorithm are named as FedProx, which deals with the heterogeneity of systems (Li et al. 2020c). Furthermore, the modifications of the aggregation algorithms are implemented as Federated Matched Averaging (FedMa), Federated Optimization (FedOpt), to mention a few. to solve different problems in FL (Wang et al. 2020; Asad et al. 2020). Besides, FL is categorized into three types as Horizontal Federated Learning (HFL), Vertical Federated Learning (VFL), and Federated Transfer Learning (FTL), based on the data distribution properties inherently used in distributed learning (Yang et al. 2019). HFL conforms to the same feature space but different samples, while VFL has the same sample ID space but is different in feature space. However, FTL has a different sample and diffident feature space, which is applied to achieve secured models (Li et al. 2020c). Currently, a couple of studies are available in the literature (Abdulrahman et al. 2021; Zhang et al. 2021; Qammar et al. 2022; Kairouz et al. 2019), that discuss the thought, structure, and relevant research work of FL. The most well-known FL applications are used in Natural Languages Processing (NLP), in banks as fraud detection models, recommendation systems to improve personalization, health care, and in many other areas (Xu et al. 2020; Chen et al. 2020; Long et al. 2020; Li et al. 2020a; Liu et al. 2021; Yang et al. 2020).

### Attacks to Federated Learning

**Fig. 4:**
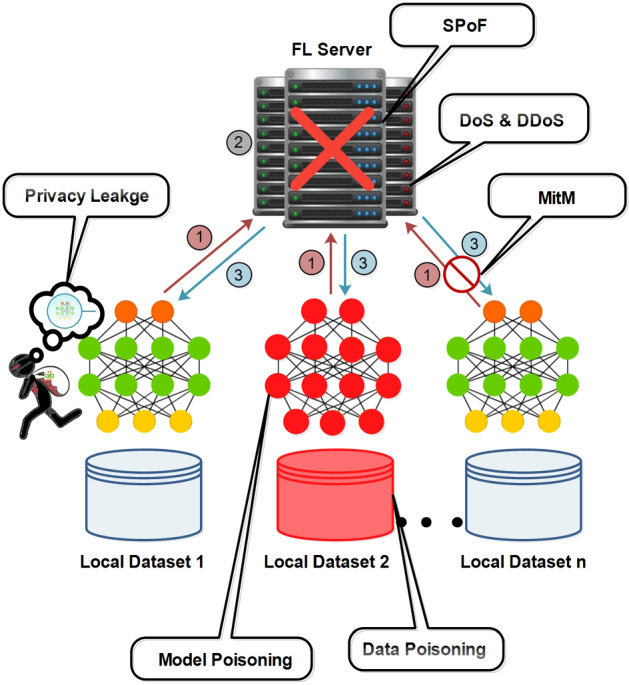
Attacks to federated learning architecture

This section explains existing attacks in the federated learning architecture and provides the answer to RQ1. Figure 4 presents the different types of attacks that lead to the failure of the entire system such as SPoF.

#### Single point of failure attack

The traditional FL structure is heavily dependent on a centralized server. In the FL system, the central server aggregates local model updates from participating devices into a fully trained global model and maintain it. In various situations, the central server can comprise the security of the FL system such as (1) instability of the central server leads to system crash (2) a compromised central server generates a false global model and (3) maximum consumption of system resources. Hence, it is vulnerable to a single point of failure (SPoF) attack (Feng et al. 2021; Li et al. 2021b). Defending against a SPoF attack from the master aggregator is challenging and promises a fully convergent model with high accuracy.

#### Denial of service and distributed denial of service attack

Malicious participants in FL model training have a different purpose instead of abolishing the model training. For instance, by continuously propagating fake model updates, malicious devices can stress the system so much that it crashes, which is called a Denial of Service (DoS) attack. Similarly, if an FL server is compromised, it repeats this process and paralyzes the entire FL system, which is referred to as a Distributed Denial of Service (DDoS) attack. Furthermore, malicious FL server or participants can add weak noise to the original global model to replace it with a new model that causes an insignificant difference in accuracy.

#### Free-riding attack

Machine learning (ML) model training requires expensive system resources such as CPU, network bandwidth, processing power, time, and many others. In the FL model training task, high cost induces dishonest participants to gain incentives without contributing to local model updates. For instance, free-riders send fake or similar model updates with minimum noise and can directly upload the untrained model. Hence, this situation in FL systems potentially leads to fairness and trustworthiness issues. Furthermore, it is difficult to detect free-riders and original data owners because they send similar model updates (Fraboni et al. 2021a).

#### Poisoning attacks

The poisoning attacks are categorized into two types i.e. data poisoning and model poisoning. By making changes to the model’s training data, the data poisoning attack is launched and the false model updates are propagated. Furthermore, malicious participants can flip the labels of datasets and implement the predefined poisoned model updates which degrade the performance of the global FL model. Therefore, data poisoning attacks ultimately lead to model update poisoning attack. Besides, reverse and random model poisoning attacks are also generated in FL systems. In random and reverse poisoning attacks, the model is updated by arbitrarily generated gradients and the training model is updated in opposite direction (Chen et al. 2018; Li et al. 2021c).

#### Man-in-the-middle attack

A Man-in-the-Middle (MitM) attack occurs between the communication of the FL server and the FL client. In this attack, the attacker pretends to be an FL server or client to send fake model updates and control the traffic. The common types of MitM attacks are session hijacking and Internet Protocol (IP) spoofing. In session hijacking, the attacker hijacks a legitimate session between a trusted FL client and the FL server. Whereas IP spoofing relates to convincing the FL server or clients that they are in connection with a trusted entity, however in reality the attacker is acting on the other side.

#### Eavesdropping Attacks

The eavesdropping attack, in the FL system, causes to leak of sensitive information about FL participants such as gender, profession, location, etc. (Wang et al. 2019a). Similarly, an adversary can delete, modify, corrupt, or intercept the broadcasted model between the FL server and participants. So far, eavesdropping attacks are considered more harmful as they can escalate to severe cyber-attacks (e.g., jamming and DoS). The jamming attack against FL systems can maliciously interrupt the network communication on the server or client end through collisions or interference.

### Overview of Blockchain

Nowadays, blockchain technology is a cutting-edge term with a lot of promise in various applications. Blockchain technology is known for the decentralized ledger technology to keep an immutable record of transactions. It has a chain of blocks that contains the transaction record, timestamp, and hash value of the associated block. The transactions stored in the blockchain are digitally signed and the hash is stored to retrieve the information for next time. In this way, the history of all transactions can be recorded in a tamper-proof manner. Furthermore, the blocks are connected in a Peer-to-Peer (P2P) network and maintain the cloned version of the integral transactions logs (Zheng et al. 2017). Blockchain is broadly categorized into three types: public or permissionless blockchain, private or permissioned blockchain, and consortium blockchain (Niranjanamurthy et al. 2018). In a public blockchain, there is no dominant authority and no party has more power than others in the network. Participants can enter and exit at any time according to their wish. Similarly, any participant can validate the transaction due to its public nature. In Bitcoin, for example, miners can validate the transactions and receive Bitcoins as rewards. With a private blockchain, a centralized structure is followed, where a single entity has full power to validate the transactions and make decisions. The private blockchain is more efficient, easy to implement, utilizes fewer energy resources, and is faster compared to the public blockchain. Besides, with the consortium blockchain, not every member has the same permissions. A few members of the blockchain network are assigned certain privileges to validate the new blocks. Other members can also validate but must reach a consensus before implementation. Different consensus algorithms are implemented depending on the requirements and environment. Consensus algorithms are the core of blockchain and determine how it will work. It is the critical technology that describes the security and improves the performance of blockchain. A consensus algorithm means an agreement, used in a decentralized network communally to collectively make a decision when it is needed. Its properties include non-repudiation, authentication, decentralized control, transparency, and byzantine fault tolerance (Seibold and Samman 2016). Authors (Xiao et al. 2020), elaborated the five components of the consensus algorithm: (1) block proposal, (2) block validation, (3) information propagation, (4) block finalization, and (5) incentive mechanism. In addition, famous consensus algorithms are such as Proof of Work (PoW), Proof of Skate (PoS), Proof of Existence (PoE), Proof of Authority (PoA), etc. Another term smart contracts (Khan et al. 2021) are deployed in blockchain as a digital agreement between two or many other parties. Based on its pre-defined function, it can store, process information, and write outputs. To prevent tampering, smart contracts are copied to each node in the blockchain. Besides, a smart contract enables transaction traceability in FL as well as irreversibility (Huang et al. 2018).

### Characteristics of blockchain-based federated learning

In this subsection, characteristics of blockchain-based federated learning versus the traditional federated learning system in response to RQ2 are described. Table 2 explains the key characteristics such as decentralization, traceability, incentives, trust, immutability, integrity, and reliability. Decentralization ensures the model updates are stored in multiple locations instead of a single location. In traditional FL systems, a single central server is used to store a trained model. If the central server crashes, the entire FL system stops working, leading to a SPoF attack. This situation incurs the imprecise model updates by falsifying all local model learnings. However, federated learning leveraging blockchain technology can resolve aforementioned issues. In the work of (Feng et al. 2021; Kim et al. 2020), the authors introduced the blockchainedFL (BlockFL) architecture to enable decentralization and secure model storage.

**Table 2:** Blockchain-based federated learning characteristics over conventional FL

Characteristics	Federated learning	Blockchain-based federated learning
Decentralization	Traditional FL systems have centralized servers that can be compromised by a malicious user and insecure	It has multiple decentralized servers that can store model updates in an irritability resistance nature and hard against a single point of failure attack
Traceability	FL does not record the history of model updates, it only stores the latest model. So accountability and audit of participants are impossible	Blockchain-based FL keeps the history of all blocks linked into a chain. The participants cannot deny the authorship of model updates.
Immutability	It is highly possible to temper historical model training updates by the malicious server which makes it difficult to detect	Tempering of records in blockchain-based federated learning is detectable and blocked by the server. Each block contains a unique hash value to make it permanent and unalterable
Incentives	The quality of local model updates are directly proportional to the global model accuracy, FL system does not have a reward mechanism to encourage participants to take part in the model training process	Participants are attracted through rewards or incentives mechanisms, in that way they contributed with quality data model updates, resulting in an accurate global model
Integrity and reliability	In federated learning, the model training process is coordinated by a single central server. The data could be corrupted by the malicious participant or a server	All blocks are connected cryptographically, in case of data alteration they can be detected easily. Blockchain proves as an inherently secure and reliable technology
Trust	A federated learning system does not provide any consensus algorithm or design an agreement for model training	Blockchain-based federated learning makes use of a consensus algorithm to establish trust between parties. The participants who agree to the contract are allowed to participate in training rounds

Similarly, blockchain-based FL provides traceability and immutability in order to track history or make model updates tamper-proof. In a blockchain network, model updates are stored through the timestep feature. The timestep in the blockchain is implemented to trace the model updates and history (Dai et al. 2019). Generally, the FL global model entirely depends on the local model updates that are stored on the central FL server. To check the local model updates shared by the client devices, the traceability property must be applied. The inclusion of traceability helps in detecting malicious endpoints and also leads to fast model convergence with better performance. Moreover, the information or model updates stored in blocks are immutable which means information cannot be changed. All the blocks are connected and store the reference hash value of the previous block. In case of an adversary can temper any block data, the hash value of the block will change and the deception of data will be easily detected. Hence, this process of assigning hash values leads to the immutable feature of the blockchain (Khan et al. 2021).

Furthermore, blockchain offers incentives to motivate the FL participating devices in model training. Blockchain promotes the incentives policy based on the contribution ratio of participants to local model updates. Hence, without any compensation devices are reluctant and less willing to participate in the training round of the traditional FL process. Apart from that, blockchain-based federated learning ensures the integrity, reliability, and robustness of the system (Wu et al. 2020a).

Integrity relates to the participation of honest clients in the FL global model training process, who are committed to assigning the smart contract. In blockchain-based federated learning, trust conforms to the two characteristics such as liveliness and loyalty. Liveliness means participated clients must keep alive during the model training rounds and continuously participate in the activities in the FL system. Similarly, loyalty relates to the contribution of local model updates to keep the FL model training process stable. However, care should be taken to optimize the use and allocation of computing resources, as end-users are reluctant to participate in model training rounds due to limited resources.

## Integrating blockchain into federated learning

Blockchain technology can be adopted into federated learning systems to embrace its characteristics as elaborated in Sect. 3.4 Moreover, Sects. 4.1 and 4.2 discuss the blockchain-based federated learning architecture and the workflow of local model updates to store and retrieve the global model from blockchain, respectively.

### Blockchain-based federated learning architecture

**Fig. 5:**
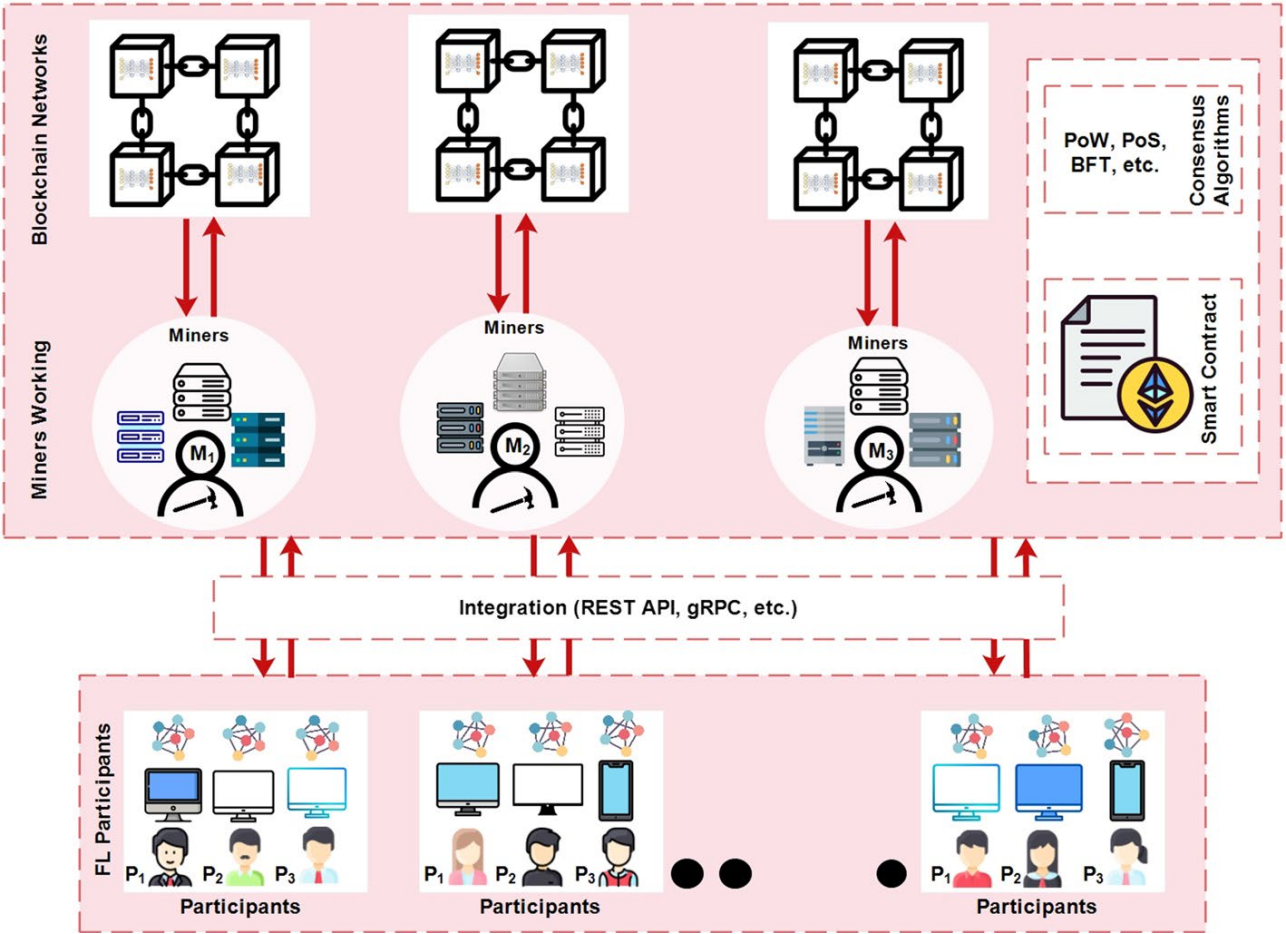
Blockchain-based federated learning architecture

Blockchain served as a fully decentralized and secure architecture for FL systems. The main objective behind the integration of blockchain is to protect the privacy of data owners, reward participants according to their contributions, and prevent malicious clients. Fig. 5 depicts the blockchain-based architecture for federated learning with its five basic components (1) FL participants, (2) FL integration with blockchain, (3) miners working, (4) smart contract, (5) consensus algorithm, and (6) blockchain network.

1. Federated learning participants Participants work as an entity or devices as in a traditional FL environment. FL participants take part in model training and send local model updates to the next phase for verification and aggregation. At first, the initial model is sent to all participating clients in the FL system. Then FL participants generate local model updates based on their raw datasets. FL participants and miners are directly communicate with each other.
2. FL integration with blockchain The integration act as middleware that provides interaction between FL participants and blockchain. Authors (Martinez et al. 2019) used the REST-API (Representational state transfer-Application Programming Interface) to interact with the Hyperledger Fabric blockchain to record and incentivize gradients uploads. Furthermore, gRPC API facilitates data transfer between FL clients and the Ethereum blockchain network using remote procedure calls (RPC) developed by Google.
3. Miners working The miners can be personal computers, standby servers, or cloud-based nodes if they willingly download the mining software. At this step, the FL participants send the local model updates to the miners. Each of the FL participant/data holders is directly connected with the miner and ensures constant communication. The miners are responsible for receiving the local model updates from participating FL devices or participants. Furthermore, aggregation is performed based on the consensus algorithm and a block is uploaded to the blockchain network.
4. Smart contract The Smart Contract (SC) in the blockchain system opens new doors for decentralized applications and automatically executes the program logic when they meet the pre-defined conditions. All conditions are transparent and immutable to participated FL clients, and before they join the FL model training process, they will agree on them. Furthermore, SC allows the clients to codify agreements without any trusted third party. Researchers (Khan et al. 2021) used smart contact in different ways such as registering the participants, coordinating the model training, aggregating the local model updates, evaluating the participants’ contribution, and awarding rewards. In Fig. 5, smart contract is assigned between FL participants and miners.
5. Consensus algorithm In the blockchain network, the consensus algorithm serves as the backbone and plays a significant role in validating transactions. All parties establish a common agreement that defines how a new block is formed, verified, and accepted on a blockchain network. As miners reach the consensus mechanism such as Proof of Work (PoW), Proof of Stake (PoS), Byzantine Fault Tolerance(BFT), to name a few, then a new block is appended into the blockchain. By adopting blockchain technology in federated learning, it becomes more flexible. FL participants will start a new FL training process, and through a consensus algorithm, miners reach an agreement to build a fully converged global model. With successful execution of consensus algorithm, block is added into the blockchain network.
6. Blockchain network Finally, verified new blocks are added to the blockchain network. The FL model process continues until it reaches the required learning rate. After that, FL clients or other participants can request to download the global model for their purposes. Finally, global model can be downloaded by the miners and FL participants can get model from them.

For instance, researchers integrate blockchain into FL in order to achieve security, accountability, and rewards (Kang et al. 2020b; Lo et al. 2022; Toyoda et al. 2020). Researchers (Toyoda et al. 2020), use blockchain to provide the rewards policy for FL participating clients who participate in the model training process. A full-fledged reward mechanism based on the contest theory is also developed. The conditions for participation in the FL training round are applied to clients, and their contribution is evaluated to assign rewards. Furthermore, the criteria for participation in the training task, the amount of reward, and the number of workers who can receive a reward are worked out. Due to the decentralization concept of blockchain, the authors deployed it to overcome the SPoF problem and proved a reliable selection of workers in federated learning. In particular, the authors (Kang et al. 2020b) selected the trusted workers to defend against malicious model updates. To select reliable workers for FL, a reputation metric is introduced based on their historic performance and recommendation. In the work of (Lo et al. 2022), a blockchain-based trusted FL architecture is proposed to introduce the accountability function. Moreover, a smart contract is designed to enable accountability which leads to an analysis of malicious FL workers. Similarly, a weighted fair algorithm is presented to improve the fairness of model training data. Consequently, the approach shows feasible performance in accountability and fairness compared to traditional FL settings.

### Workflow of blockchain-based federated learning architecture

**Fig. 6:**
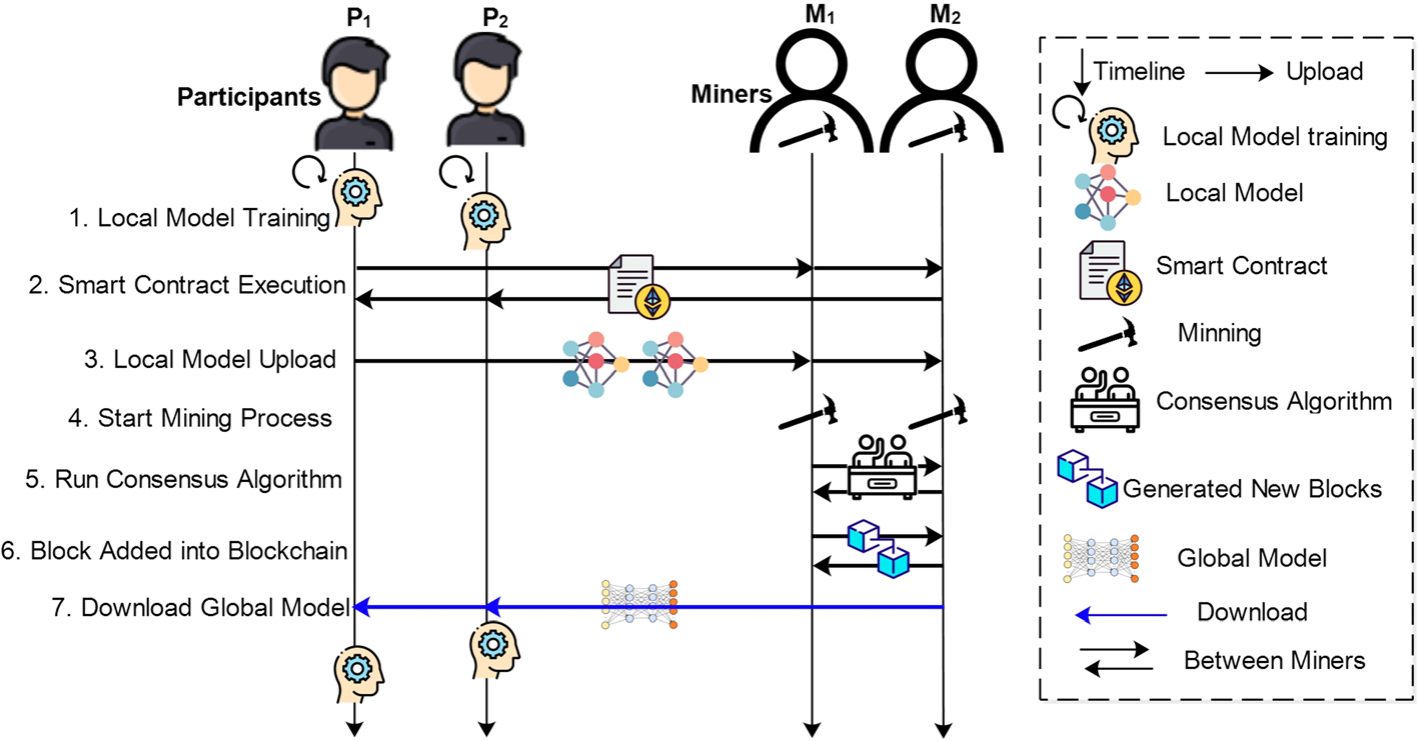
One-epoch workflow of blockchain-based federated learning system

The one-epoch operation of the blockchain-based federated learning is depicted in Fig. 6 with its seven steps. These steps are repeated until the global model has converged fully or reached the appropriate learning rate.

1. Local model training At the initial step, FL clients train the local model updates based on their local datasets and upload the model for further procedures such as verification, aggregation, to mention a few.
2. Smart contract execution The smart contract is executed between parties to interact with the blockchain network. For instance, FL participants register through the smart contract when they fulfill the required criteria for the FL model training process. After the successful registration of FL participants, the local model updates are transferred to the miners.
3. Local model upload The local model updates are uploaded to the miners on the blockchain. The miners verify and authenticate the local model updates based on the consensus protocol.
4. Start mining process The associated miners receive the local model updates from the registered FL participants. Then the miners verify the local model updates and also aggregate them.
5. Run consensus algorithm Each miner runs the consensus algorithm until it receives a newly generated block from other miners. The new block is then broadcasted to all miners in the network.
6. Add block into blockchain Finally, a new block is added to the blockchain network.
7. Download global model Devices can request to download the global model. FL participant devices can download the model freely as they utilized their resources to train the model. While on the other hand, external devices have to pay charges to access the global model. In this way, the entire community can benefit from fully trained models.

In a blockchain network, the blocks are connected in a distributed and decentralized nature that contains the hash of the previous block, information about model updates, timestamps, etc. Hence, the record is stored permanently and immutably. In blockchain storage, there are typically two types: (1) on-chain storage that all the records are stored in one ledger, and (2) off-chain storage, where data is stored in another third-party system. Due to the limited size of blocks for data storage in the blockchain, only the unique identity of the entire data needs to be stored. The complete data streams are stored in third-party storage such as InterPlanetary File System (IPFS) (Benet 2021). The IPFS is a decentralized and private storage system that allows the permanent storage of data. In the literature (Kumar et al. 2020), the authors implemented the IPFS to store actual models and the hash value send to the blockchain to guarantee immutability. Furthermore, for future use, the hash values are retrieved from the IPFS to identify relevant model updates. Similarly, (Yuan et al. 2021) proposed to use the IPFS to store files to upload and download a model from IPFS in training rounds. However, only the unique identity of each model parameter is stored in the blockchain.

### Blockchain deployment frameworks

The famous blockchain deployment frameworks used in recent studies of blockchain-based federated learning namely Ethereum, Hyperledger Fabric, Financial Blockchain Shenzhen Consortium (FISCO) Be Credible, Open and Secure (BCOS), Corda, and Enterprise Operating System (EOS) are discussed in this section. Different blockchain frameworks have distinguished properties. For instance, public blockchain offers consistent performance, private blockchain offers robust security, while consortium blockchain offers more customization options. After a thorough literature review, Table 3 describes the blockchain frameworks with key features such as blockchain category, smart contracts with applied language, consensus algorithms, and level of support for FL as implemented in literature work.

#### Ethereum

Ethereum is a decentralized, open-source blockchain framework that allows users to create smart contracts. Formally, Ethereum is permissionless blockchain platform, launched in 2015, deployed the Proof-of-Work (PoW) consensus algorithm, and has a native cryptocurrency known as Ether (Buterin 2013). Furthermore, Ethereum allows smart contracts implementation written in Solidity language. In this context, the authors (Vaikkunth Mugunthan 2020) used Ethereum based smart contracts in BlockFlow architecture which provides a secure FL system through model updates. Additionally, other frameworks such as Baffle (Ramanan and Nakayama 2020) and ChainFL (Korkmaz et al. 2020) run on Ethereum enabled FL systems and smart contracts used for model aggregation and update process in FL.

#### Hyperledger fabric

Hyperledger fabric is a permissioned blockchain hosted by the Linux Foundation. It is used to implement distributed applications written in languages such as Go and Java. The smart contracts in Hyperledger Fabric are known as chain codes to automatically execute the application logic. Furthermore, consensus protocols including Practical Byzantine Fault Tolerance (PBFT) and Raft are used and it has no fundamental cryptocurrency (Androulaki et al. 2018; Zhao et al. 2021) implemented a decentralized model training infrastructure for federated learning using the Hyperledger fabric, which is more secure and robust as compared to a centralized structure. Smart contracts are applied to reach the aggregation in the training process in an open and transparent manner to ensure integrity and safety. Additionally, in the work of (Zhang et al. 2020), the authors used the blockchain algorithm for secure communication of model updates between server and FL clients. The information about a required global model can be searched on the blockchain and then the current model is transmitted to the network.

#### EOS.IO

The Enterprise Operating System (EOS) blockchain was developed to compete with the Ethereum blockchain framework. EOS is the first leading system that provides high throughput by Delegated Proof of Stake (DPoS) algorithm and uses in decentralized applications. The smart contract in EOS.IO is written C++, which was later augmented by WebAssembly also known as Wasm (Huang et al. 2020). For instance, the authors (Martinez et al. 2019) presented the plan to implement the EOS-based federated learning system where clients can benefit through incentives, leading to robust and efficient model performance. Similarly, another author (Kang et al. 2020a) introduced the scalable EOS-based decentralized FL system to detect poisoning model updates and apply the Proof of Verifying (PoV) consensus protocol.

#### FISCO BCOS

Financial Blockchain Shenzhen Consortium (FISCO), a leading consortium blockchain, was founded by WeBank with the participation of Tencent and Huawei. FISCO is not a single blockchain, but a unique blockchain application designed to benefit the general public. Additionally, it is a secure, portable blockchain and supports PBFT and Raft consensus algorithms (BCOS 2018). Researchers (Li et al. 2020b) proposed a novel committee consensus protocol for blockchain-based federated learning to mitigate malicious model updates and improve system scalability and incentive mechanism.

#### Corda

Corda was created in 2014 by the R3 consortium as an open-source and permissioned blockchain framework. Corda underlines data privacy and follows the “Know Your Customer” term to share the transactions across the network. The smart contracts are written in Java and Kotlin language to support decentralized applications (Brown 2018). In research (Kang et al. 2019), the authors implemented Corda V4.0 in the training process for federated learning models to determine the fairness of workers sending the useful model updates. Similarly, the reputation metric is considered and calculated through a consensus protocol, which relies on the reputation score and work to gain rewards.

**Table 3:** Comparative analysis of blockchain deployment frameworks

Blockchain framework	Category	Consensus algorithm	Smart contract language	Hosted by	Cryptocurrency	Level of support for FL	Related studies
Ethereum	Public	PoW	Solidity	Ethereum developers	Ether (ETH) and Bitcoin (BTC)	High	Buterin (2013), Vaikkunth Mugunthan (2020), Ramanan and Nakayama (2020), Korkmaz et al. (2020)
Hyperledger fabric	Private	PBFT	GoLang, Java	Linux Foundation	None	High	Androulaki et al. (2018), Zhao et al. (2021), Zhang et al. (2020)
EOS.IO	Public and consortium	DPoS	C, C++	Block.One	EOS	Moderate	Huang et al. (2020), Martinez et al. (2019), Kang et al. (2020a)
FISCO BCOS	Consortium	PBFT, Raft	Solidity, C++	Webank	None	Moderate	Li et al. (2021b), BCOS (2018)
Corda	Consortium	PBFT, Raft	Kotlin, Java	R3 Consortium	None	Moderate	Brown (2018), Kang et al. (2019)

## State-of-the-art: blockchain-based federated learning approaches

**Fig. 7:**
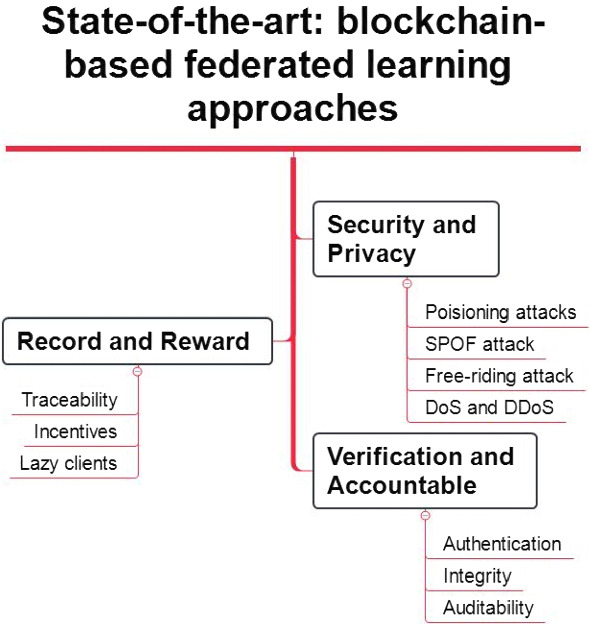
State-of-the-art: blockchain-based federated learning approaches

Formally, FL is a kind of machine learning to train the model on local devices and then aggregate the model on the central server. Therefore, model training performance and security are the critical aspects to be considered. This section elaborated the answer to RQ3: What are the state-of-the-art blockchain-based federated learning approaches in security and privacy, records and rewards, and verification and accountability to secure the traditional FL system? Figure 7 illustrated that blockchain-based FL approaches work against various attacks and provide traceability and accountability to ensure FL security. Accordingly, state-of-the-art blockchain-based federated learning approaches are introduced in the following subsections to provide improvements in model training.

### Blockchain-based approaches to security and privacy in federated learning

Blockchain-based decentralized approaches mitigate the security and privacy attacks in the FL environment. In the literature, blockchain-based FL approaches are elaborated to deal with SPoF, poisoning, free-riding and DDoS attacks. Table 4 elucidates the relevant studies with respect to major contributions, blockchain implementation frameworks in federated learning systems, consensus algorithm, and block structure.

The authors presented the BytoChain (Li et al. 2021c) framework based on blockchain technology to provide security and privacy in federated learning systems. The structure of Bytochain is divided into three parts: (1) data owners that send trained local model updates, (2) verifiers that verify the model updates, (3) miners that aggregate the model, and (4) task publishers add the global model into the blockchain network. The verifiers in the BytoChain are able to minimize the workload of miners in sense of verification overhead and works in parallel manners. In addition, a consensus algorithm named Proof of Accuracy (PoA) is applied to effectively detect the privacy loss. It also works well against security attacks such as Denial of Service (DoS), reverse model poisoning, and free-riding attacks. Consequently, BytoChain achieves equal accuracy under attack settings as FL without attacks. Another framework called ChainsFL (Yuan et al. 2021) builds on the two layers of blockchain and federated learning. The main-chain and sub-chain of blockchain are made up of Raft and Direct Acyclic Graph (DAG), respectively. The raft-based blockchain is liable for coordinating the devices in order to complete model training tasks with substantial computation and high storage capabilities. Furthermore, DAG or tangle consensus is applied to deal with interaction with the subchain layer. ChainsFL effectively detected fake model updates and lazy clients. Accordingly, performance metrics such as convergence and robustness of the ChainsFL are compared with FedAvg (McMahan et al. 2017) and Asynchronous FL (AsynFL) (Cong Xie 2019). The extensive experiments show that ChainsFL successfully detected and eliminated the malicious devices and model updates.

Ma (2020) proposed a blockchain-assisted decentralized FL (BLADE-FL) framework to prevent the model from malicious learning updates as well as SPoF attacks. The BLADE-FL framework consists of three layers: (1) the network layer ensures task publishing and trains the nodes, (2) the blockchain layer provides tracking and aggregation of model updates, and 3) the application layer uses the smart contract (SC) to execute the FL events. After training the global model, the task publisher provides incentives to the participants who participated in the training round with benign model updates. Similarly, miners are also rewarded for successful aggregation and broadcasting of the model. Moreover, BLADE-FL deals with privacy, resource allocation, and lazy participants issues. Kang et al. (2020a) introduced a blockchain-enabled federated edge learning (BFEL) method with a decentralized server. A consortium blockchain is deployed with a Proof of Verifying (PoV) consensus algorithm to identify poisoning model updates and verify the quality of the updates. Miners are selected based on the highest computation and storage resources to implement a consensus algorithm. Moreover, miners with insufficient resources are eliminated in real-time. Besides, gradient leaks from inference attacks are reduced through a gradient compression scheme. Finally, BFEL ensures the model training flexibility, malicious model update detection, and overcoming computation overhead.

In the work of Short et al. (2020), researchers implemented blockchain technology to deal with security issues in FL. The algorithm is implemented in the smart contract, can run external tools, and keeps the privacy of datasets from clients. For the experiment, a private blockchain tool such as Hyperledger fabric is used to fulfill the requirements of blockchain-enabled federated learning. Results show that the proposed algorithm works well against poisoning attacks. The authors (Zhao et al. 2021) proposed the blockchain-based FL committee (BFLC) consensus algorithm to guard against malicious attacks and reduce computation overhead. BFLC framework is divided into three steps: (1) blockchain storage, (2) committee consensus algorithm, and (3) model training. In blockchain storage, two different types of blocks are generated to store local and global model updates, respectively. The consensus algorithm verifies the gradient updates and assigns scores to them before adding them to the blockchain. Furthermore, model training involves a certain number of local model updates and then aggregates into a global model after verification. Finally, profit sharing by contribution scheme is implemented to motivate the participants in the model updates process. BFLC performs best under malicious attacks and minimizes the transmission cost. Kumar et al. (2020) presented the decentralized training for FL with blockchain to enable security and incentive mechanism. For security purposes, Differential Privacy (DP) and Homomorphic Encryption (HE) techniques are performed. Similarly, Elastic Weight Consolidation (EWC) is applied to enhance the operation of a global model. Eventually, experiments prove that blockchain deployment via Ethereum and IPFS enables the fully decentralized model training in FL with improved security and privacy features.

Shayan et al. (2021) introduced Biscotti, a decentralized peer-to-peer (P2P) scheme based on blockchain and the exchange of secrets as verifiable random functions (VRFs) to maintain privacy and security between FL peers. A consensus protocol, proof-of-federation (PoF) is combined with the multi-Krum defense (Blanchard et al. 2017) and differential privacy (DP) to protect against poisoning and Sybil attacks. Besides, PoF provides protection against groups of colluding peers that overcome the system without enough stake ownership. The central node in federated learning leads to privacy and SPoF attack that results in the failure of the entire system. In this perspective, Fed-BC (Wu et al. 2020b) is presented as a blockchain-based decentralized federated learning framework to integrate robustness and privacy. For experimental purpose, the blockchain implementation is built by Hyperlegdger fabric and IPFS deployed as a decentralized storage. Eventually, a deep neural network (DNN) is used to train the FL model with two hidden layers, and a number of ten clients participated in the training round.

**Table 4:** Blockchain-based federated learning security and privacy approaches

Approaches	Major contribution	Blockchain type	Block structure	Block storage	Consensus algorithm	Blockchain tool
BytoChain (Li et al. 2021c)	Byzantine resistant consensus Proof of Accuracy (PoA) Detected the random and reverse poisoning, overfitting poisoning, DoS, and free-riding attacks	Private	Merkle Tree	–	PoA	–
Chainsfl (Yuan et al. 2021)	Raft and DAG-based blockchain consensus algorithm Synchronous and asynchronous learning combined to dismiss the drag down of stragglers	Private	Merkle Tree	Off chain	Raft and DAG	Hyperlegdger fabric
BLADE-FL (Ma 2020)	Prevented from the Single point of failure (SPoF) attack Misbehaved and lazy participants are recognized	Public	–	–	PoW	–
BFEL (Kang et al. 2020a)	Proof of Verifying (PoV) consensus algorithm to filter out poisoning updates A gradient compression scheme with PoV	Public and Consortium	Merkle Tree	–	PoV, DPoS, and PBFT	EOS.IO
(Short et al. 2020)	Based on the accuracy improvement, model updates are evaluated Traceability function of blockchain for the detection of malicious users	Private	–	–	–	Hyperlegdger fabric
BFLC (Li et al. 2021b)	Committee consensus algorithm to reduce model poisoning attacks Storage optimization, scalability of BFLC, and incentives	Consortium	–	On-chain	Committee	FISCO
(Kumar et al. 2020)	Differential privacy (DP) and homomorphic encryption (HE) to improve the security in FL Incentive scheme	Public	–	Off-chain	–	Ethereum
Biscotti (Shayan et al. 2021)	Prevent Sybil and poisoning attacks using VRF and PoF, and multi-krum, respectively Implemented the secret sharing scheme for secure model aggregation	Private	Merkle Tree	Off-chain	PoF	Hyperlegdger fabric
Fed-BC (Wu et al. 2020b)	Fully decentralized system avoids SPoF attack and privacy leakage	Private	–	Off-chain	–	Hyperlegdger fabric

### Blockchain-based federated learning record and reward approaches

The self-interested workers or data holder devices in FL model training are reluctant to participate unless they receive financial compensation. However, previous studies have shown that devices contribute their resources conclusively in federated learning, which is not an ideal approach as the cost is encountered in model training (Kumar et al. 2020; Zhou et al. 2019). Furthermore, untrusted participants in FL can perform malicious action by sending malicious model updates which lead to model poisoning attacks. By tracing or recording the model updates, malicious actions can be detected and these participants can be punished. Accordingly, a reliable participant can be motivated through rewards to send benign model updates. Consequently, well-designed approaches are required to measure the participants’ beneficial contributions and then announce the rewards for them. In Table 5, a summary of blockchain-based federated learning approaches are discussed which highlights record and reward schemes for participating workers in model training rounds to motivate them.

Fedcoin (Liu et al. 2020), the approach is presented with blockchain to incentivize FL participants to update the model. The concept of Shapley Values is implemented in previous studies for profit distribution. But, the SVs calculation process is more time-consuming and computationally complex. In Fedcoin, SVs are defined as proof of Shapley (PoSap) protocol with blockchain consensus algorithm to provide an incentive to FL participants with non-repudiation. Furthermore, the authors launched the demonstration system which performs FL tasks in real-time and awards based on their performance. Martinez et al. (2019) proposed a record and reward approach by evaluating the participants’ contributions in the model training process. Through blockchain, model updates are tracked, recorded, and rewarded based on computation power cost utilized by FL participants. A Class-Sampled Validation-Error Scheme (CSVES) is introduced to validate the valuable model updates for rewarding via a smart contract. Consequently, participants received incentives for model updates and ensure more robust FL models.

Kang et al. (2019) introduced reputation as a fair metric to evaluate the robustness and trustworthiness of participants in FL systems. For this purpose, a reputation-aware participant selection scheme is designed by using blockchain technology. Blockchain has the properties of non-repudiation and resilience to enable honest reputation management of workers in updating FL models. Besides, the incentive approach is combined with reputation metrics to encourage devices to send high-quality data for model training. In the end, experiments are applied to real datasets and accurate reputation calculation of devices is achieved, which greatly improves model accuracy.

Implementing smart contracts on a blockchain network leverages transparent, independent, and immutable features. In this context, the authors (Behera et al. 2021) have used the smart contract based on the Ethereum blockchain to incentivize the FL participants. The intuitive contribution of participants is measured and associated with the model training as well as the rewards process. Similarly, in Batool et al. (2022) authors introduced a monetization scheme based on blockchain for FL clients along with a multi-dimensional auction named as FL-MAB. The clients are selected concerning their resources including data size, bandwidth, and relative rewards when submitting their bid. Moreover, blockchain-based federated learning provides non-repudiation, integrity, and encouraged the clients with cryptocurrency as a reward.

**Table 5:** Blockchain-based federated learning record and reward approache

Approaches	Major contribution	Blockchain type	Block structure	Block storage	Consensus algorithm	Blockchain tool
FedCoin (Liu et al. 2020)	PoSap consensus protocol for fair payment distribution between clients Record of all payments	Public	Merkle Tree	–	PoSap	–
(Martinez et al. 2019)	Class-Sampled Validation-Error Scheme (CSVES) for rewarding and validating the model updates Record model training updates	Private	–	Off-chain	–	EOS
(Kang et al. 2019)	Reputation metric to measure the fairness of model updates Workers reputation is calculated and managed Encouraged the high reputation workers with effective incentives	Consortium	–	On-chain	PBFT	Corda V4.0
(Behera et al. 2021)	Record contributions of clients through smart contract and then rewarded A decentralized communication scheme for FL	Consortium blockchain setup	Merkle Tree	Off-chain	–	Ethereum
FL-MAB (Batool et al. 2022)	Measured the relative contribution of every client by Shapley value, and allocate rewards accordingly	Public	–	Off-chain	–	Ethereum

### Blockchain-based federated learning verification and accountable approaches

Verification and accountability approaches are introduced to prevent the attackers from sending malicious model updates. Blockchain-based FL approaches uses smart contracts to detect and financially penalize the attackers. Additionally, lazy clients send malicious model updates or replace the original model with a fake or less precise model to save computational cost. Hence, to rectify the security of FL, it is mandatory to implement the verification procedure that ensures the integrity and authenticity of model updates during the training process to prevent malicious attacks. The blockchain-based approaches in federated learning perform verification and accountability of model updates. In this case, an immutable feature of blockchain provides data provenance through traceability of the FL training procedure. Similarly, blockchain-based FL verification schemes are presented in the Table 6, to build trust and improve security.

VFChain (Peng et al. 2021) refers to the verifiable and auditable FL approach by using blockchain technology. To establish verifiability, a committee selection scheme is introduced to aggregate the model updates and record verified updates in the blockchain. In the case of auditability, a data structure named Dual Skip Chain (DSC) is presented for blockchain to support the search and rotation of committees in an authenticated and secure way. Furthermore, an optimization method is introduced to provide multiple model training tasks. Finally, extensive experiments have demonstrated that VFChain effectively performed verifiability and auditability in FL through blockchain technology. Awan et al. (2019) offered the privacy-preserving FL approach using blockchain, which comprises three elements: (1) server, (2) clients, and (3) aggregators. To record local and global model updates a distributed immutable ledger is implemented to ensure tamper resistance. By tracking the model transactions, the trust and verification mechanism is provided in blockchain-based federated learning. Moreover, the tracking process measures each client’s contribution to model updates and rewards schemes. Similarly, in Desai et al. (2021), the authors have developed an accountable FL method to distress attackers. In the BlockFLA framework, attacks are detected through accountability with hybrid blockchain technology such as public and private tools leads to Ethereum and Hyperlegdger fabric, respectively. The public architecture of blockchain-based FL is implemented to execute intensive algorithms and can be retrieved by anyone. Moreover, private blockchain ensures communication efficiency and deals with sensitive data to alleviate data leakage. Accordingly, to evaluate the BlockFLA, a FedAvg and SignSGD (stochastic gradient descent) algorithms are implemented with various features including parallelism.

Moreover, Lo et al. (2022) proposed a trustworthy federated learning framework empowered with blockchain to improve accountability and equality in FL systems. A smart contract and weighted fair data algorithm are designed for the data model registry to enable accountability and fairness, respectively. For evaluation, a COVID-19 X-ray dataset is employed and accomplished a better performance in terms of accuracy as compared to vanilla federated learning settings. In the same way, BlockFLow (Vaikkunth Mugunthan 2020) ensures the accountability and privacy for federated learning systems in decentralized manners. The model auditing process evaluates the good or malicious behavior of model contributors. Furthermore, after the auditing process, contributors are rewarded with cryptocurrencies based on the public Ethereum blockchain. Evaluation results show that subsequent auditing scores reflect the quality of honest and malicious participants.

**Table 6:** Blockchain-based federated learning verification and accountable approaches

Approaches	Major contribution	Blockchain type	Block structure	Block storage	Consensus algorithm	Blockchain tool
Vfchain (Peng et al. 2021)	A VFChain to verify and audit the updates Aggregated models and proofs recorded by committee selection	Private	Dual Skip Chain	–	–	Hyperlegdger fabric
BC-based PPFL (Awan et al. 2019)	An accountable method to record local and global model updates Tracking of data flows in FL system provides the trust and verification	Private	–	Off-chain	PoW, PoS	Hyperlegdger fabric
BlockFLA (Desai et al. 2021)	Through accountability protects against adversarial attacks Discouraged the backdoor attacks and applied the transparency	Hybrid	–	Off-chain	PoW, PBFT	Hyperlegdger fabric, Ethereum
(Lo et al. 2022)	A trustworthy system to enable accountability in FL. For auditing purposes track the local model and global model. To improve the fairness of data and models a weighted fair training was introduced	Parity consortium blockchain	–	Off-chain	Proof-of-Authority (PoA)	Galaxy FL framework (Ethereum)
Blockflow (Vaikkunth Mugunthan 2020)	A unique accountability mechanism for model contribution Resultant auditing scores reflect the quality of the honest and malicious clients	Public	–	Off-chain	–	Ethereum

## Discussion

To our knowledge, this is the first systematic literature review on blockchain-based federated learning. The results of SLR indicate that integrating blockchain into federated learning can solve most of the attacks that have occurred in conventional FL, given the architecture, workflow, and characteristics of blockchain-based FL. Furthermore, researchers (Li et al. 2021c; Kang et al. 2020b; Shayan et al. 2021) implemented the blockchain-based FL to mitigate the security attacks and worked as a decentralized system. Similarly, blockchain technology introduced the reward scheme in federated learning, where FL participants share the local model updates, the updates are first verified by miners, and then they receive rewards based on their contribution (Liu et al. 2020; Batool et al. 2022). In addition, blockchain-based FL has traceability, immutability, and accountability features to record, maintain the model history and punish the FL participants, respectively (Peng et al. 2021; Desai et al. 2021; Lo et al. 2022). However, there are still a couple of challenges that exist in blockchain-based FL (see Sect. 7.1 for details).

Blockchain requires the majority of network for PoW, the stake of cryptocurrency as PoS, and a permissioned network for an honest consensus mechanism that leads to the correct execution of smart contracts and provides immutability. Ethereum-based smart contracts are implemented in Solidity language where the complexity of execution is measured in terms of the gas price to be paid for each transaction. This prevents infinite loops and promotes fair competition for constrained storage and computing power. However, blockchain faces challenges of storage and high energy consumption for PoW. Off-chain computation and storage are recommended to address scalability and storage issues but verifying malicious participants in a system is problematic. Some future directions are provided (see Sect. 7.2) to address the privacy issues on Ethereum, authentication of FL participants, miner selection, and smart contract vulnerabilities and management.

## Open issues and future directions

### Open issues

1. *Malicious miners in blockchain-based FL* In blockchain-based federated learning, miners perform a significant role in terms of model aggregation and reach the consensus algorithm to get the reward. To increase the profit, malicious miners detect the vulnerabilities in incentive distribution mechanisms. Therefore, exploiting the mining behavior leads to degradation of the honest miners’ revenue and has a serious impact on the mining pool, resulting in pool mining attacks. Researchers have discussed this attack in previous studies (Eyal and Sirer 2014; Sapirshtein et al. 2017), however, unfortunately, malicious miners have not elaborated in the context of blockchain-based federated learning systems.
2. *Miners selection in blockchain-based FL* In blockchain-based federated learning architecture, the honesty of the miners validates the secure and privacy-preserving models. The authors (Alladi et al. 2020) presented the two types of miners: static and dynamic (or moving) miners. Static miners use the fiber-optic network to communicate with end devices for model update transactions. Dynamic or moving miners using the wireless network for interaction in terms of sharing model parameters must be carefully planned. Hence, miners’ selection, network resources consumption, and secure design must be considered in the future.
3. *Dark side of immutable storage of FL models* The immutability feature of the blockchain ensures that transactions are stored permanently. In blockchain-based federated learning systems, model updates are stored forever and in a tamper-proof manner. Model updates and transactions cannot be altered by any involved party or others. Although it is a great advantage of blockchain, it has a dark side as well. In case of an error in transactions, it cannot be rectified. Similarly, in terms of smart contract assignments between parties are unchangeable. If both parties are agreed upon the changes, due to the blockchain immutability feature they cannot. Another kind of limitation is that a smart contract has irreversible nature, once a smart contract is implemented, it cannot be altered. Furthermore, any tries to hack the model and access it for legal or illegal purposes are stored in the blockchain permanently.
4. *Exploitation of smart contracts* A smart contract ensures the execution of the logic originally written into it. After the execution of the logic, the final state is stored into the network immutably. However, the faulty implementation of smart contracts does not guarantee security. Investigation of existing smart contracts reveals the vulnerabilities and security issues they present. The most common vulnerabilities are indirect execution of unknown code and incorrect exception handling. Due to the activation fallback function in smart contracts, for instance, parameter type confusion can occur when a developer invokes the contract. Furthermore, in Solidity smart contract exceptions are thrown and cannot be handled in the same procedure. Exceptions are handled through the collaboration between contracts. The contracts can be exploited by adversaries, if exceptions are not resolved correctly then the transactions are rolled back.
5. *Vulnerabilities in blockchain frameworks* In subsection 4.3, blockchain frameworks implemented in federated learning are elaborated. The EOS.IO blockchain framework is developed to compete with Ethereum. No doubt, EOS.IO achieved higher performance throughput and was more efficient (Larimer 2018). However, security vulnerabilities and attacks have emerged in recent years. Consequently, millions of dollars were lost from attacks (Berman 2018; Street 2019). Similarly, the authors (Mitra 2019) detected bugs in Ethereum smart contracts and copy-paste vulnerabilities are also present to analyze.
6. *Malicious end-devices* In the federated learning model training process, various end devices are participated to train the model and send local model updates. Malicious devices can inject poisoned or tempered model data that leads to a poisoned global model. As a result, the entire aggregation process is compromised and the outcome results are less accurate and consume extra resources. The trusted and authenticated end devices are required for securing FL model convergence.
7. *Asynchrony of end-devices* In FL model training, various devices may enter or exit the process at different times. This affects the efficiency and accuracy of the global model. End-devices may drop out from the training process for various reasons, such as network problems, defective devices, minimum memory capacity, etc. Furthermore, the asynchrony issue leads to the unbalanced distribution of rewards and affects the accuracy of a global model.
8. *Synchronization Issue:* FL systems run in a synchronous manner in which the central FL server waits for all local model updates, then start other training round and aggregate all updates. Hence, model convergence speed slows down due to lazy participants. As they consumed prolonged time to finish the one training iteration.
9. *Blockchain Forking Issue:* Forking occurs when a block is mined simultaneously by multiple miners. In competition based techniques, blocks are added to the chain before the consensus protocols, and higher scalability ultimately leads to a higher chance of forking. Similarly, misconducted miners utilized the insufficient computing power of the system which results in blockchain forks (Gemeliarana and Sari 2018). Despite that, a customized PV (Probabilistic Verification) scheme can be applied to counter and mitigate the forking (Liu et al. 2019).

### Future direction

The integration of blockchain technology into federated learning is a promising research direction, as it provides significant features of security and privacy models. Furthermore, this integration enables the implementation of a recording and reward mechanism with accountability characteristics. However, future directions are still required in order to solve open issues.

1. *Authentication scheme for blockchain-based FL* To recognize the end-devices in the FL system the authentication scheme should be implemented. The devices should be registered to get unique IDs before participating in FL model training. In blockchain-based federated learning, the authentication scheme can be possible with device registration. Similarly, it is crucial to develop frameworks to select devices that do not send fake or unreliable model updates for federated learning systems.
2. *Vulnerabilities in smart contracts* To detect vulnerabilities in smart contracts static analysis should be performed before implementing in blockchain-based FL systems. Moreover, smart contracts should be secure through code auditing, analysis, and review. They should be tested against vulnerabilities. Static analysis through automated tools can provide comprehensive details and verification of fixes issues (Blaize 2021). Other frameworks such as ZEUS (Kalra et al. 2018) can also be used for smart contract verification and optimality required robust security tools.
3. *Mechanism for Miners’ selection and verification* Miners are responsible to add new blocks to a blockchain network. Given that malicious miners can add falsification results to the block and can gain incentives from other honest miners. It is suggested to propose mechanisms for miners’ selection and verification. It is possible to choose the leader of miners based on performance and participation in the blockchain-based federated learning system. The leader should also perform some additional roles regarding miner selection, miner registration, miner verification, authentication, etc. In the case of selected miners, model updates are verified, models are downloaded and aggregated.
4. *Privacy to Ethereum blockchain-based FL* Zero-knowledge proofs (ZKPs) technologies can add privacy to Ethereum Blockchain. Authors (Ben-Sasson et al. 2018), introduced ZKPs via scalable transparent argument of knowledge (STARKs). For future research in blockchain-based FL, it is recommended to implement ZKPs via STARKs to improve proof creation performance, post-quantum security, and eradicating the need for a trusted setup.
5. *Life cycle of contract management* The use of contract management tools can solve the problems of immutability and irreversibility. By dealing with the life cycle of contract management, these limitations can be eliminated. A contract management solution Fabasoft contracts (Fabasoft 2021), which provides functions for storing contracts in an audit-proof form, is used in Europe. Additionally, it provides ready-to-use contract management schemes, automatic modeling of rights, and verification.

To be successful in blockchain-based federated learning systems, certain efforts are required in terms of data resources, aligned motivation, and clear goals between companies. In some researches (Kang et al. 2019), financial rewards with digital currencies are announced. The mentioned scheme is not enough to motivate whole companies to participate in federated learning systems. Consequently, from this perspective, additional development schemes of models and prevalent adoption of cryptocurrencies are a prerequisite.

## Conclusion

The integration of blockchain technology into FL architecture provides decentralized, secure, and robust solutions, as blocks are connected in the form of a chain. Deployment of smart contracts makes them immutable and maintains the history of model updates. More precisely, in this paper, we have elaborated the basic description of the ecosystem of blockchain and federated learning. The potential issues that exist in the inherited structure of FL i.e. single point of failure attack, distributed denial of service attack, man-in-the-middle attack, etc. are investigated in context to answer the RQ1: What are the potential security and privacy attacks in traditional federated learning which can be solved by blockchain technology? Then the blockchain properties are comparatively studied how they can be integrated into FL and successfully secure the FL environment to justify RQ2: What are the promising characteristics of blockchain for federated learning to provide a secure environment? The blockchain-based federated learning architecture with its entire mechanism, workflow, and deployment framework are presented. Blockchain substantially improves the FL efficiency, security, privacy, and is also able to implement the incentive mechanism in order to answer the RQ3. Furthermore, blockchain-based FL approaches in the view of security, rewards, and accountability are presented. Based on a systematic literature review, open issues are investigated to clarify the RQ4. Eventually, future research directions are identified to answer the RQ5: What are promising future research directions for effectively implementing blockchain technology in federated learning? We hope that this paper will lead to the development of a robust blockchain-based federated learning system that manages the open issues.
